# Neuroinflammation associated with proviral DNA persists in the brain of virally suppressed people with HIV

**DOI:** 10.3389/fimmu.2025.1570692

**Published:** 2025-05-21

**Authors:** Sarah J. Byrnes, Janna Jamal Eddine, Jingling Zhou, Emily Chalmers, Emma Wanicek, Narin Osman, Trisha A. Jenkins, Michael Roche, Bruce J. Brew, Jacob D. Estes, Thomas A. Angelovich, Melissa J. Churchill

**Affiliations:** ^1^ ATRACT Research Centre, School of Health and Biomedical Sciences, RMIT University, Melbourne, VIC, Australia; ^2^ Department of Infectious Diseases, The University of Melbourne at the Peter Doherty Institute for Infection and Immunity, Melbourne, VIC, Australia; ^3^ Department of Neurology and Immunology, Peter Duncan Neuroscience Unit, St Vincent’s Hospital, University of New South Wales, Darlinghurst, NSW, Australia; ^4^ University of Notre Dame, Sydney, NSW, Australia; ^5^ Vaccine & Gene Therapy Institute, Oregon Health & Science University, Beaverton, OR, United States; ^6^ Life Sciences, Burnet Institute, Melbourne, VIC, Australia; ^7^ Department of Microbiology, Monash University, Melbourne, VIC, Australia; ^8^ Department of Medicine, Monash University, Melbourne, VIC, Australia

**Keywords:** HIV, brain, neuroinflammation, reservoirs, microglia, astrocytes

## Abstract

Despite viral suppression with antiretroviral therapy (ART), people with HIV (PWH) continue to exhibit brain pathology, and ~20% of individuals develop HIV-associated neurocognitive disorders. However, the state of cellular activation in the brain of virally suppressed (VS) PWH and the impact of local viral reservoirs on cellular activation are unclear. Using multiplex immunofluorescence imaging, here, we demonstrate that the frontal cortex brain tissue from both non-virally suppressed (nVS; n=17) and VS PWH (n=18) have higher frequencies of astrocytes and myeloid cells expressing interferon-inducible Mx-1 and proinflammatory TNFα relative to HIV-seronegative individuals (p<0.05 for all). The frequency of TGF-β1+ cells were also elevated in the brain tissue from both nVS and VS PWH, which may support active immunoregulatory responses despite ART. Importantly, the frequency of Mx1+ myeloid cells correlated with levels of total HIV DNA and intact and 5′ defective HIV proviral DNA (p<0.05 for all) in the brain of VS PWH. These findings demonstrate that cell activation persists in the brain of VS PWH and is associated with HIV DNA in the brain, which may contribute to neuropathology.

## 1 Introduction

Although sustained treatment with antiretroviral therapy (ART) suppresses HIV plasma viremia, which limits the risk of acquired immunodeficiency syndrome or viral transmission, virally suppressed people with HIV (VS PWH) continue to have chronic tissue damage and an elevated risk of developing comorbidities and long-term non-AIDS-related pathology (1–5). Specifically, VS PWH have a higher incidence of brain atrophy (6), reduced synaptic density (7), and elevated clinical neurometabolites associated with cellular activation than age-matched people without HIV with approximately 20% of VS PWH developing neurocognitive disorders (3, 6, 8). The mechanisms driving neuropathology and/or cognitive disorders are unclear; however, viral persistence in the central nervous system (CNS) and peripheral tissues, and ongoing neuroinflammation and systemic inflammation penetrating the brain are all thought to play fundamental roles (3, 9).

We and others have recently demonstrated that a reservoir of HIV DNA persists in the brain tissue of VS PWH, primarily in the frontal cortex (10–13). Levels of intact and defective HIV proviral DNA present in the CNS did not differ between VS and non-virally suppressed (nVS) PWH, demonstrating that ART does not reduce the size of the viral reservoir in the frontal cortex, which may impact cell activation.

Chronic HIV infection is associated with heightened measures of neuroinflammation and immune activation as primarily measured by surrogate markers in plasma and/or cerebrospinal fluid (CSF) (14–16). Studies, including our own, have utilized models of chronic ART-treated simian immunodeficiency virus (SIV) infection to further demonstrate chronic immune activation in the brain parenchyma at a cellular level (17–19). Specifically, we found heightened type I interferon (IFN), oxidative stress, and transforming growth factor (TGF-β1) signaling pathways in the frontal cortex of SIV+ non-human primates (NHPs) despite long-term viral suppression with ART (18). Whether these markers of immune activation are similarly elevated in the CNS of VS PWH, and importantly, the role of viral persistence in the brain on cellular activation is unclear.

In this study, cellular activation in the frontal cortex of the brain from VS PWH was measured using quantitative spatial multiplex immunofluorescence imaging of the autopsy brain tissue. The relationship between the local viral reservoir in the brain and neuroinflammation were examined to further understand the mechanisms driving neuroinflammation in VS PWH.

## 2 Materials and methods

### 2.1 Cohort

Formalin-fixed paraffin-embedded (FFPE) and matched fresh frozen human autopsy frontal cortex tissue from PWH and HIV-seronegative individuals were generously provided by the National NeuroHIV Tissue Consortium (NNTC, USA, https://nntc.org). The median (IQR) post-mortem interval (PMI) was 8.50 (5.75–16.5) h. Exclusion criteria included extended post-mortem interval (>27 h), any known co-infections, and comorbidities associated with the brain or vascular system. Tissue was not specifically anatomically matched within the frontal cortex. Clinical information including ART regimen, CD4+ T cell counts, plasma, and CSF viral loads were provided unless stated (**Table 1**). CNS penetrance scores were calculated as previously described (20).

**Table 1:** Clinical parameters.

ID	Age	Sex (% male)	Plasma VL	CSF VL	CD4	ART	CPE score	VS (years)	Average T-Score
HIV seronegative									
HIV -ve 1	51	F	–	–	–	–		–	–
HIV -ve 2	61	M	–	–	–	–		–	–
HIV -ve 3	^a^	M	–	–	–	–		–	–
HIV -ve 4	39	M	–	–	–	–		–	–
HIV -ve 5	^a^	F	–	–	–	–		–	–
HIV -ve 6	^a^	F	–	–	–	–		–	–
*Median* *(IQR)*	51 (39-61)	50%							
Non-virally suppressed									
nVS PWH 1	56	M	61,223	^a^	24	3TC/ABC, ATV, BIC/FTC/TFV	13	–	34.6
nVS PWH 2	43	M	64	^a^	110	DRV, EFV/TFV, RTV	8	–	45.9
nVS PWH 3	57	F	576,000	10,005	98	None	–	–	39.6
nVS PWH 4	39	F	2,857	^a^	757	None	–	–	40.1
nVS PWH 5	48	M	750,000	^a^	2	None	–	–	^a^
nVS PWH 6	38	F	39,184	^a^	1	None	–	–	^a^
nVS PWH 7	56	F	35,723	^a^	73	BIC/FTC/TFV	9	–	43.1
nVS PWH 8	47	F	367,620	314	2	None	–	–	54.8
nVS PWH 9	62	M	222,840	501	8	3TC, ABC, D4T	7	–	26.2
nVS PWH 10	37	M	287,947	220	3	3TC, D4T, EFV	7	–	34.1
nVS PWH 11	49	M	750,000	1,101	3	None	–	–	50.4
nVS PWH 12	40	F	157,009	408	5	EFV/FTC/TDF	7	–	^a^
nVS PWH 13	55	F	17,387	UD	8	None	–	–	36.1
nVS PWH 14	42	M	688	^a^	441	3TC/ABC	5	–	61
nVS PWH 15^b^	35	M	2,827	78	211	3TC, D4T, IDV	7	–	43.9
nVS PWH 16^b^	46	M	17,500	^a^	^a^	^a^	^A^	–	^a^
nVS PWH 17^b^	57	F	730,085	^a^	25	None	–	–	34.6
*Median* *(IQR)*	47 (40–56)	52.9%	61,223 (17,387–367,620)	361 (184.5–651)	16 (3–101)		7 (7–8.25)		40.1 (35.6–45.9)
Virally suppressed									
VS PWH 1	67	M	UD	UD	355	3TC/ABC, ATV, RTV	8	7.32	28.9
VS PWH 2	46	M	UD	^a^	61	DRV, EFV/TFV, RTV	8	3.76	46.4
VS PWH 3	57	M	UD	^a^	48	3TC/ZDV, EFV	9	0.75	39.4
VS PWH 4	59	F	UD	^a^	328	EFV/TFV, RGV	7	2.47	^a^
VS PWH 5	39	M	UD	^a^	112	DRV, EFV/TFV, RTV	8	1.46	47.5
VS PWH 6	62	M	UD	UD	274	3TC, ABC, RGV	8	13.6	^a^
VS PWH 7	64	F	UD	^a^	537	3TC/ZDV, EFV	9	2.89	38.1
VS PWH 8	50	M	UD	105	113	ATV, EFV/TFV, RTV	7	5.30	^a^
VS PWH 9	52	M	UD	UD	417	EFV/FTC/TDF	7	6.04	54.1
VS PWH 10	64	M	UD	^a^	798	3TC, ABC, EFV	8	2.54	39.5
VS PWH 11	63	M	UD	UD	140	3TC, EFV/TFV	6	9.15	^a^
VS PWH 12	66	M	UD	UD	527	EFV/FTC/TDF	7	10.0	28.6
VS PWH 13	58	M	UD	^a^	119	3TC, EFV/TFV	6	1.42	39.1
VS PWH 14	62	M	UD	UD	133	FTC/TDF, NVP	8	7.75	^a^
VS PWH 15	58	F	UD	^a^	155	3TC/ABC, DTG	9	1.46	39.8
VS PWH 16	62	M	UD	^a^	172	DTG, FTC/TDF	8	3.93	^a^
VS PWH 17	52	M	UD	^a^	383	3TC, D4T, EFV	7	2.58	^a^
VS PWH 18	61	F	UD	^a^	25	3TC/ABC, ATV, RTV	8	6.89	41.6
*Median* *(IQR)*	60 (53.3–62.8)	77.8%	49 (40–50)	50 (50–55)	163 (114.5–376)		8 (7–8)	3.8 (2.49–7.21)	39.6 (38.6–44)

3TC, lamivudine; ABC, abacavir; ATV, atazanavir; BIC, bictegravir; CD, cluster of differentiation; CI, cognitive impairment; CPE, central nervous system penetration effectiveness; CSF, cerebrospinal fluid; D4T, stavudine; DRV, darunavir; DTG, dolutegravir; EFV, efavirenz; F, female; FTC, emtricitabine; IDV, indinavir; M, male; IQR, interquartile range; nVS, non-virally suppressed; NVP, nevirapine; RGV, raltegravir; RTV, ritonavir; TDF, tenofovir disoproxil fumarate; TFV, tenofovir; UD, undetectable (<60 HIV RNA copies/m:L); VL, viral load; VS, virally suppressed; ZDV, zidovudine. Cognitive impairment: T score >40.

a Missing data.

b Non-virally suppressed individuals with HIV associated encephalitis.

### 2.2 Quantification of cellular immune activation in human brain tissue

Multiplex fluorescent immunohistochemistry was performed as previously described (21) with the following amendments: FFPE tissue was deparaffinized and rehydrated prior to antigen retrieval and hydrogen peroxide treatment. Tissues were then incubated in the first primary antibody, either Mx1 (1:100; 2 h; cat: MABF958; Merck, Rahway, NJ, United States), TNFα (1:100; 2 hours; cat: ab1793; Abcam), or TGF-β1 (1:50; overnight; cat: ab215715; Abcam, Waltham, MA, United States). Primary antibody was detected with the anti-rabbit/mouse polymer HRP-conjugated system (cat: DET-HP1000; EMD Millipore, Burlington, MA, United States). Opal fluorophore (opal 570; 1:200; cat: FP1488001KT; Akoya, Marlborough, MA, USA) was used to visualize the first primary antigen. To remove residual antibody for the next round of antigen detection, each slide was boiled for 20 min in citrate (pH6) retrieval buffer and left to cool at room temperature. This method was repeated for the second primary antibody (CD68; 1:200; 2 h; cat: M0814; DAKO, Jena, TH, Germany) and the third primary antibody (GFAP; 1:5,000; 2 h; cat: Z0334; DAKO, Jena, TH, Germany) and visualized with Opal 650 (1:200; cat: FP1496001KT; Akoya, Marlborough, MA, USA) and Opal 520 (1:200; cat: FP1487001KT), respectively. Nuclei were counterstained with DAPI (1:750; cat: 94774; DAKO, Jena, TH, Germany), and lipofuscin was quenched with True Black (1:20 in 70% ethanol; 30 s; cat: 23007; Biotium, Freemont, CA, USA). Slides were rinsed in H_2_O and mounted with Fluoromount G (cat: 495802; Invitrogen, Carlsbad, CA, USA). Mounted slides were dried overnight and scanned at 20× magnification (Axioscan 7; Zeiss, Oberkochen, BW, Germany). Images were analyzed as a whole or stratified into gray and white matter using HALO AI 3.6 software (Indica Labs, Albuquerque, NM, USA). The percentage of positive and colocalized cells were quantified using HighPlex FL v4.2 with the frequency of marker-positive cells expressed relative to the number of nuclei per tissue section.

### 2.3 HIV DNA quantification in frontal cortex tissue

Genomic DNA was extracted from homogenized fresh frozen frontal cortex brain tissue (~10 mg pieces) and intact (Ψ+ and *env*+), 3′ defective (Ψ+ and *env*−) and 5′ defective (Ψ− and *env*+) HIV DNA standardized to RPP30 was quantified using the intact proviral DNA assay (QX200; BioRad, Hercules, CA, USA), as previously described (10).

### 2.4 Statistics

All statistical analysis was completed using GraphPad Prism software (version 10.2.2 Windows). Comparisons between groups were made using non-parametric Kruskal–Wallis tests with Dunn’s *post-hoc* analysis for multiple comparisons; median and interquartile ranges are shown. Spearman’s correlations were performed on log transformed data; rho (ρ) and p-values are shown.

## 3 Results

To characterize the cellular environment and HIV viral reservoir in the brain of nVS and VS PWH, matched fresh frozen and formalin-fixed paraffin-embedded (FFPE) frontal cortex tissue was obtained from nVS (n=17), VS PWH (n=18), and HIV-seronegative controls (HIV−; n=6) from the National NeuroHIV Tissue Consortium (NNTC, USA; https://nntc.org/; **Table 1**). Viral suppression was defined by >1.4 years of undetectable HIV RNA copies/mL in plasma. One participant (VS PWH 3) under this threshold was included, as they had long-term suppression (5.35 years) prior to two viral load tests <650 copies/mL, 0.75 years prior to death. This individual had two undetectable viral load tests prior to death, one of which was the day prior to death and a CD4 T-cell count in range of the virally suppressed group. PWH who were treated with ART but did not meet the criteria of undetectable viral loads as described above were collated in the nVS group for analysis. VS PWH were suppressed for a median of 3.8 years, were generally older than both nVS PWH (median age, 60 vs. 47 years; p<0.001) and seronegative individuals (median age, 60 vs. 51, p>0.05), and were predominantly men (77.5%). The average T score measure of cognitive impairment did not differ between VS and nVS groups (median T score: VS, 39.6 vs. nVS, 40.1; p>0.05). Three nVS PWH were classified with HIV encephalitis by expert neuropathologists at the NNTC.

### 3.1 Elevated reactive astrocytes persist in the frontal cortex of the brain in virally suppressed PWH

**Figure 1:**
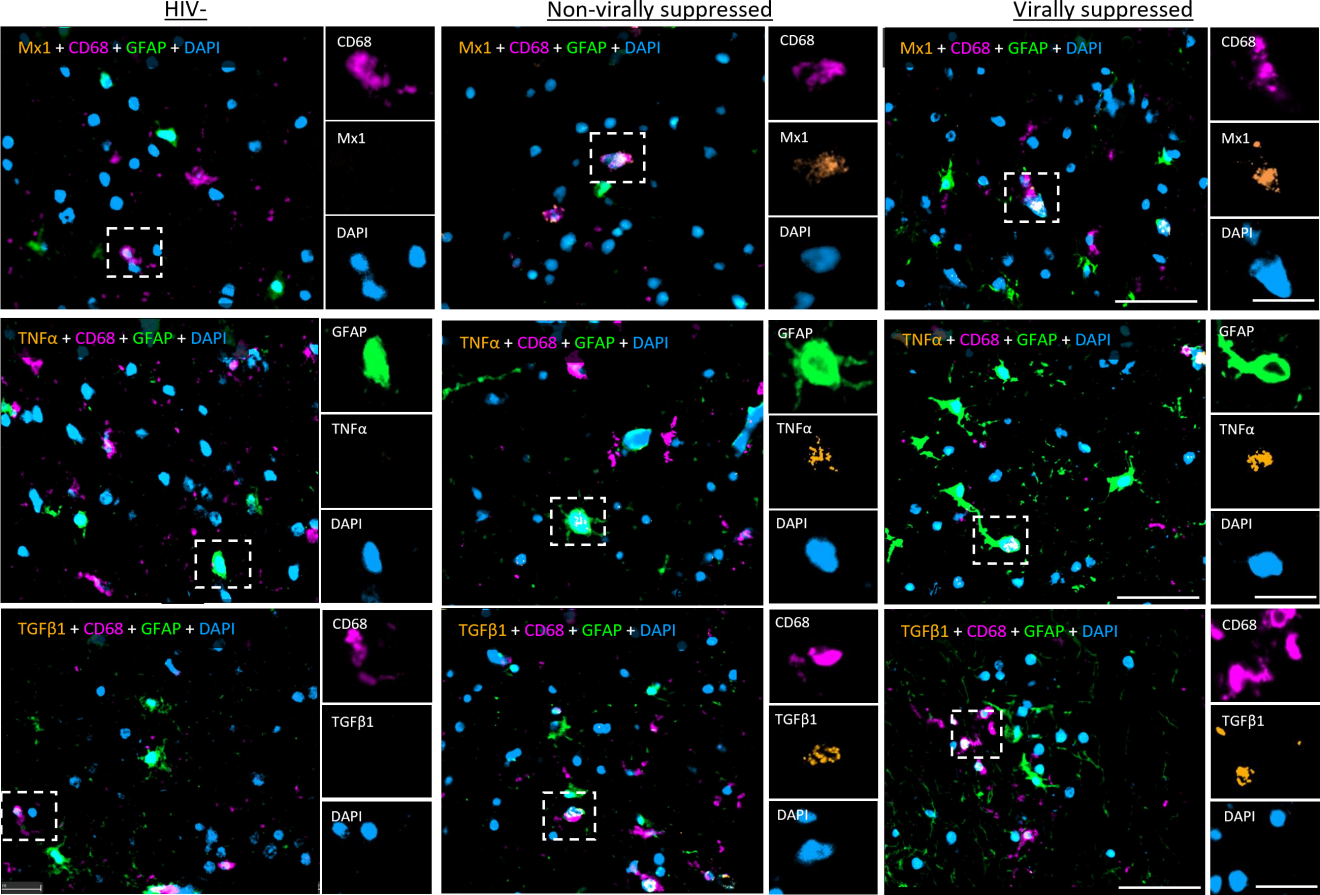
Representative images of multiplex immunohistochemistry of human frontal cortex tissue from PWH. Representative images of fluorescent IHC staining for Mx1, TNFα, and TGF-β1 (orange) multiplexed with CD68 (magenta), GFAP (green), and DAPI (blue) performed on the frontal cortex tissue of the brain from non-virally suppressed and virally suppressed PWH or HIV-seronegative (HIV−). Images shown at 30× magnification: scale bars: 50 and 20 µm (inset).

To assess immune activation in the frontal cortex of the brain from nVS and VS PWH, multiplex immunofluorescence imaging specific for type I IFN responses, proinflammatory NF-κB-mediated signaling and immunoregulatory TGF-β1 signaling were performed. Representative images of immunofluorescence staining are shown in **Figure 1**. Myeloid cells were defined as CD68^+^ and astrocytes were defined as glial fibrillary acidic protein (GFAP) positive, which are upregulated during cell activation (22, 23). Due to regional differences in cell composition and function between white matter and gray matter in the frontal cortex, each region was annotated and assessed individually and a combined total value. The frequency of CD68^+^ myeloid cells (consisting of both microglia and perivascular macrophage) did not differ between HIV-seronegative, nVS PWH, and VS PWH when examined in total frontal cortex, and in the white matter and gray matter alone (**Supplementary Figures S1A–C**). While the frequency of astrocytes in the gray and white matter were similar between groups (HIV-seronegative, VS PWH, and nVS PWH), there was a higher frequency of astrocytes in the total frontal cortex for nVS PWH relative to HIV-seronegative controls (**Supplementary Figures S1D–F**).

### 3.2 Virally suppressed PWH harbors a state of cellular activation in the frontal cortex of the brain despite ART

**Figure 2:**
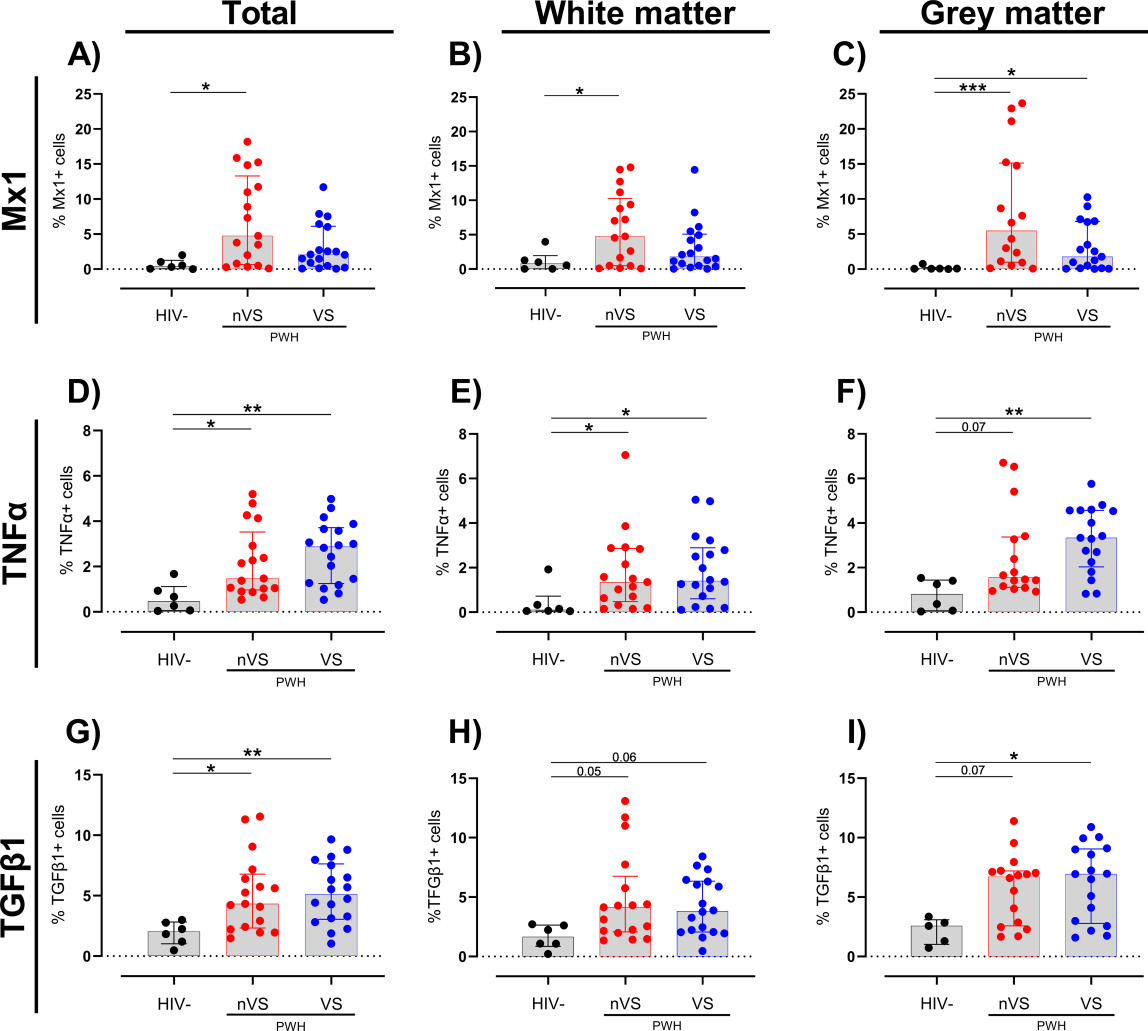
The frequency of cells expressing immune activation and immunoregulatory proteins are elevated in the frontal cortex from non-virally suppressed and virally suppressed people with HIV. The frequency of cells expressing **(A–C)** Mx1, **(D–F)** TNFα, and **(G–I)** TGF-β1 in the total (white + gray matter) and white and gray matter tissue alone of the frontal cortex from non-virally suppressed PWH (nVS; n=16–17), virally suppressed PWH (VS PWH; n=17–18), and HIV seronegative controls (HIV−; n=5–6) as measured by multiplex immunofluorescence. Data unavailable for gray matter for n=1 HIV-seronegative controls (TGF-β1 only), n=1 VS PWH and n=1 nVS PWH due to no gray matter region present in the tissue section provided. Median and interquartile ranges shown. Comparisons made using Kruskal–Wallis test with Dunn’s *post-hoc* tests (*p<0.05; **p<0.01; ***p<0.001).

Multiplex immunofluorescence staining for CD68, GFAP, Mx1, tumor necrosis factor alpha (TNFα), and TGF-β1 was performed to assess whether cellular activation is present in the frontal cortex of nVS and VS PWH. A higher frequency of cells expressing the anti-viral IFN-inducible protein Mx1 was present in total frontal cortex tissue from nVS PWH relative to HIV-seronegative individuals (**Figure 2A**). When stratifying by region, the frequency of Mx1^+^ cells were elevated in both white and gray matter from nVS PWH (p<0.05 for both). However, Mx1^+^ cells were elevated in gray matter from VS PWH (**Figures 2B, C**), suggesting that activation was localized to gray matter during viral suppression. Total frontal cortex tissue from both nVS and VS PWH also exhibited a higher frequency of cells expressing proinflammatory TNFα relative to HIV-seronegative individuals (**Figure 2D**). The frequency of TNFα^+^ cells was elevated across white matter when analyzed separately for both nVS and VS PWH (p<0.05 for all; **Figure 2E**). In the gray matter, TNFα was elevated in VS PWH (p<0.05), and an increasing trend was present in nVS PWH (p=0.066; **Figure 2F**), indicating widespread production of TNFα within the frontal cortex of the brain from PWH.

Interestingly, a higher proportion of cells expressing the immunoregulatory cytokine TGF-β1 was observed in the total frontal cortex tissue of both nVS and VS PWH compared to HIV-seronegative individuals (p<0.05, **Figure 2G**), which may indicate active immunoregulatory responses. Regional analysis identified an increasing trend of TGF-β1^+^ cell frequency in nVS and VS PWH in the white matter (nVS p=0.054, VS p=0.055; **Figure 2H**). In gray matter, TGF-β1 was increased in VS PWH (p<0.05) and was higher in nVS PWH; however, this did not reach significance (p=0.071; **Figure 2I**). Importantly, the frequency of immune markers did not correlate with age (**Supplementary Table S1**). Together, these observations demonstrate that a heightened state of cellular activation and reciprocal regulatory responses exists in the brain of PWH, which persists despite viral suppression with ART.

### 3.3 Activated myeloid cells and astrocyte phenotypes contribute to neuroinflammation in PWH

**Figure 3:**
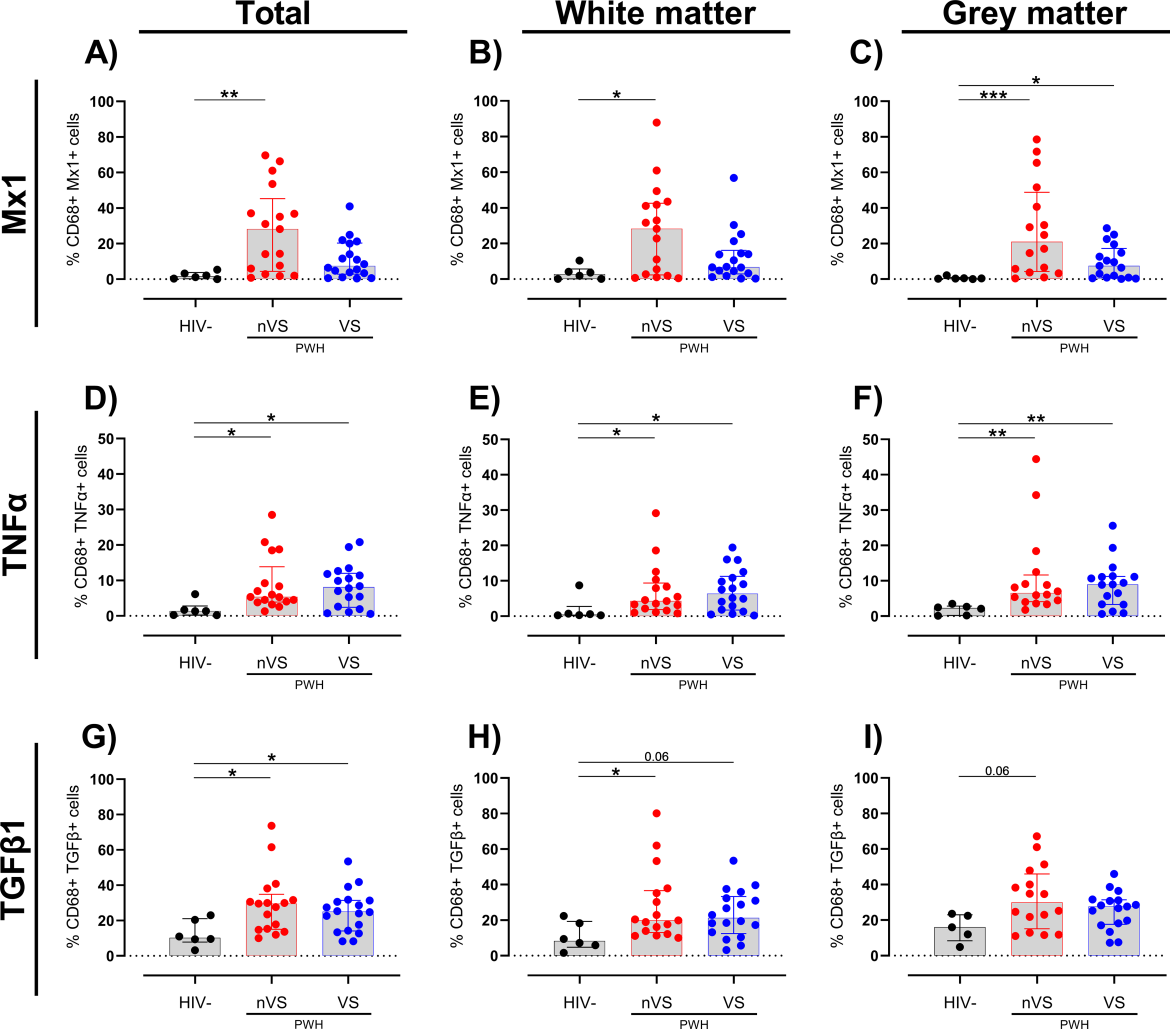
Non-virally suppressed and virally suppressed PWH have a higher frequency of myeloid cells expressing inflammatory markers in the frontal cortex. Frequency of **(A–C)** Mx1, **(D–F)** TNFα, and **(G–I)** TGF-β1 colocalized with CD68^+^ myeloid cells as a percentage of total CD68^+^ cells in total frontal cortex tissue, white matter or grey matter of non-virally suppressed (nVS; n = 16-17), virally suppressed (VS; n = 17–18) people with HIV and HIV-seronegative controls (HIV−; n = 5–6). Data unavailable for gray matter for n=1 HIV seronegative controls (TGF-β1 only), n=1 VS PWH, and n=1 nVS PWH due to no gray matter region present in the tissue section provided. Median and interquartile ranges shown. Comparisons made using Kruskal–Wallis test with Dunn’s *post-hoc* tests (*p<0.05; **p<0.01; ***p<0.001).

To further define the cellular origins of immune activation in the frontal cortex of nVS and VS PWH, colocalization analysis was performed using HALO imaging analysis software. Specifically, cell-marker-positive cells (i.e., either CD68^+^ myeloid cells or GFAP^+^ astrocytes) expressing a particular activation marker were enumerated and expressed as a percentage of cell-marker-positive cells. In the total frontal cortex and white matter, nVS PWH had a higher frequency of myeloid cells expressing Mx1 relative to HIV-seronegative individuals (p<0.05 for both; **Figures 3A, B**). Additionally, the frequency of Mx1^+^ myeloid cells were higher in both nVS and VS PWH in the gray matter (p<0.05 for both; **Figure 3C**). Additionally, the frequency of TNFα^+^ myeloid cells were elevated in total frontal cortex from both nVS and VS PWH relative to HIV-seronegative individuals (p<0.05 for both; **Figure 3D**), supporting heightened cell activation leading to broad proinflammatory cytokine production in the frontal cortex that is not restored with ART treatment. The frequency of TNFα^+^ cells were also elevated across white and gray matter for both groups (p<0.05 for all; **Figures 3E, F**), indicating a higher frequency of TNFα-producing myeloid cells across all regions of the frontal cortex in PWH. The frequency of myeloid cells producing TGF-β1 was elevated in both nVS and VS PWH (p<0.05 for both; **Figure 3G**). A higher frequency of TGF-β1 expressing myeloid cells were observed in nVS PWH in the white matter (p<0.05), and an increasing trend was present in VS PWH (p=0.058; **Figure 3H**). In the gray matter, only nVS PWH displayed a trend of elevated TGF-β1^+^ myeloid cells (p=0.061; **Figure 3I**).

**Figure 4:**
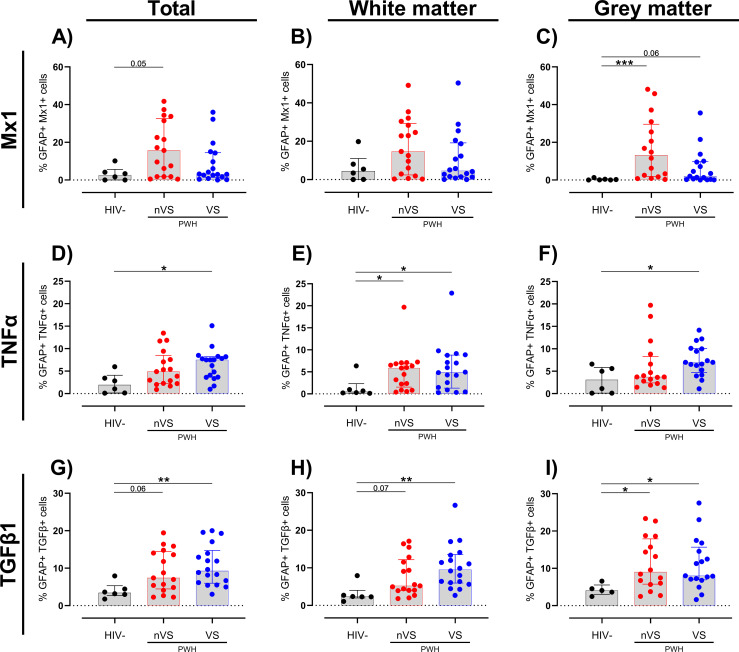
A higher frequency of activated astrocytes persists in the frontal cortex tissue from non-virally suppressed and virally suppressed people with HIV. Frequency of **(A–C)** Mx1, **(D–F)** TNFα, and **(G–I)** TGF-β1 colocalized with GFAP as a total of GFAP^+^ cells in white and gray matter in non-virally suppressed (nVS; n=16–17), virally suppressed people with HIV (VS HIV; n=17–18) and HIV-seronegative controls (HIV-; n=5-6) as measured by multiplex immunofluorescence. Data unavailable for gray matter for n=1 HIV seronegative controls (TGF-β1 only), n=1 VS PWH, and n=1 nVS PWH due to no gray matter region present in the tissue section provided. Median and interquartile ranges shown. Comparisons made using Kruskal–Wallis test with Dunn’s *post-hoc* tests (*p<0.05; **p<0.01; ***p<0.001).

Similar to observations for myeloid cells, an increased trend of Mx1^+^-activated astrocytes was present in total frontal cortex tissue of nVS PWH (p=0.054; **Figure 4A**). Sub-analysis by region demonstrated that while there were no significant changes between groups in the white matter (**Figure 4B**), nVS PWH exhibited a higher frequency of Mx1^+^ astrocytes in gray matter compared to HIV-seronegative individuals (p<0.05; **Figure 4C**). Additionally, a trend of increased frequency of Mx1+ astrocytes was present between VS PWH and HIV-seronegative individuals (p=0.060; **Figure 4C**), indicating that the gray matter is a site of elevated Mx1-expressing astrocytes in PWH.

An increase in TNFα-expressing astrocytes was observed in VS PWH (p<0.05; **Figure 4D**) relative to HIV-seronegative individuals in total frontal cortex tissue. In contrast to a consistently increased prevalence of TNFα-expressing myeloid cells across total, white, and gray matter in nVS and VS PWH (**Figures 3D–F**), a higher frequency of TNFα^+^ astrocytes in nVS and VS PWH were found in the white matter (p<0.05 for both; **Figure 4E**), while TNFα^+^ astrocytes were only elevated in VS PWH in the gray matter (p<0.05; **Figure 4F**).

Finally, the incidence of TGF-β1^+^ astrocytes were significantly higher in total frontal cortex tissue from VS PWH (p<0.05; **Figure 4G**), and a trend to a higher frequency was observed in nVS PWH (p=0.062) relative to HIV-seronegative individuals. Following sub-region analysis, a higher frequency of TGF-β1^+^ astrocytes was also observed in VS PWH (p<0.05; **Figure 4H**), and a trend was present in nVS PWH (p= 0.073) relative to HIV-seronegative individuals in the white matter. Similarly, the frequency of TGF-β1+ astrocytes were increased in both nVS and VS PWH compared to HIV-seronegative individuals (p<0.05 for both; **Figure 4I**).

### 3.4 Neuroinflammation in the frontal cortex of VS PWH is associated with total and 5′ defective HIV DNA in the brain

To determine whether cellular activation in the brain was associated with the level and type of HIV proviral DNA in the frontal cortex of PWH, the levels of intact, 3′ defective and 5′ defective HIV DNA were quantified by the intact proviral DNA assay (IPDA), and correlation analysis with measures of cellular activation was performed. Similar to our previous findings (10), the frontal cortex of all PWH in both nVS and VS groups contained similar levels of total HIV DNA (**Supplementary Figure S2A**). Intact proviral DNA was also present in the frontal cortex of both nVS and VS PWH (**Supplementary Figure S2B**) at similar levels, confirming that the brain tissue from both nVS and VS PWH harbored a reservoir of intact proviral HIV DNA that did not differ between groups. The level of total HIV proviral DNA was associated with Mx1^+^ myeloid cells in the total frontal cortex tissue and also in white and gray matter regions alone when analyzed separately (p<0.05 for all, **Table 2**). Total HIV DNA was also associated with levels of Mx1^+^ astrocytes in gray matter (ρ=0.507, p=0.040) and total frontal cortex tissue (ρ=0.510, p=0.039), with a trend observed in white matter (ρ=0.461, p=0.054; **Table 2**), supporting a relationship between levels of HIV proviral DNA and Mx1-expressing cells in the frontal cortex of VS PWH. Sub-analysis for intact, 3′ defective and 5′ defective proviral DNA, as measured by IPDA, demonstrated that levels of intact HIV proviral DNA correlated with those of total Mx1^+^ cells in total frontal cortex and gray matter (p<0.05 for both), Mx1+ myeloid cells in all regions (p<0.05 for all) and Mx1^+^ astrocytes in both total frontal cortex and gray matter (p<0.05 for both; **Table 2**). Importantly, levels of 5′ defective HIV DNA were associated with Mx1^+^ myeloid cells in total frontal cortex, white and gray matter, and GFAP+ cells in total and white matter tissue (p<0.05 for all, **Table 2**), supporting an association between 5′ defective HIV proviruses in the brain of ART-suppressed PWH with Mx1-expressing myeloid cells and astrocytes in the frontal cortex tissue. Conversely, levels of 3′ defective HIV DNA did not correlate with Mx1, TNFα, or TGF-β1 expression in either white or gray matter (p>0.05 for all; **Table 2**). Total or intact HIV proviral DNA did not correlate with TNFα or TGF-β1 expression (p>0.05 for all; **Table 2**), indicating an alternative viral independent driver of cells expressing these proteins in VS PWH. Together, these findings indicate that the level of total and specifically 5′ defective HIV DNA in the frontal cortex tissue was associated with activation of anti-viral type I IFN signaling in VS PWH.

**Table 2:** Association between HIV DNA in the frontal cortex and neuroinflammation in virally suppressed PWH.

	Mx1+ cells		TNFα+ cells		TGF-β1+ cells		Mx1+ myeloid		Mx1+ GFAP	
Grey matter	Rho	p-value	Rho	p-value	Rho	p-value	Rho	p-value	Rho	p-value
Intact HIV DNA	**0.577**	**0.017**	0.009	0.975	−0.221	0.391	**0.575**	**0.018**	**0.618**	**0.010**
3’ defective HIV DNA	0.113	0.666	−0.382	0.131	−0.284	0.268	0.098	0.708	0.115	0.660
5’ defective HIV DNA	0.463	0.063	−0.213	0.410	−0.306	0.231	**0.538**	**0.021**	0.466	0.062
Total HIV DNA	0.463	0.063	−0.086	0.744	−0.297	0.247	**0.523**	**0.026**	**0.507**	**0.040**
White matter										
Intact HIV DNA	0.418	0.084	0.187	0.458	−0.007	0.977	**0.522**	**0.026**	0.385	0.115
3’ defective HIV DNA	0.212	0.399	−0.300	0.226	−0.269	0.280	0.152	0.548	0.189	0.453
5’ defective HIV DNA	**0.562**	**0.015**	0.110	0.663	−0.152	0.548	**0.651**	**0.003**	**0.523**	**0.026**
Total HIV DNA	**0.498**	**0.035**	0.148	0.559	−0.189	0.453	**0.606**	**0.008**	0.461	0.054
Total Frontal Cortex										
Intact HIV DNA	**0.550**	**0.018**	0.090	0.722	−0.107	0.673	**0.572**	**0.013**	**0.488**	**0.040**
3′ defective HIV DNA	0.168	0.505	−0.432	0.073	−0.313	0.206	0.127	0.625	0.218	0.499
5′ defective HIV DNA	**0.554**	**0.017**	−0.051	0.842	−0.199	0.428	**0.510**	**0.039**	**0.507**	**0.040**
Total HIV DNA	**0.525**	**0.025**	−0.007	0.977	−0.222	0.376	**0.512**	**0.038**	**0.510**	**0.039**

Mx1, MX dynamin like GTPase; TGF-β1, transforming growth factor beta; TNFα, tumor necrosis factor alpha.

p-value and rho determined by non-parametric Spearman correlation (p<0.05 statistically significant).

Bold values indicate significance (p<0.05); underlined values indicate trend (p>0.05, <0.1).

## 4 Discussion

Despite viral suppression with ART, PWH continue to develop comorbidities at a greater rate than seronegative individuals that significantly impact health and daily function. Neurocognitive issues and neuropathologies affect ~20% of VS PWH, reportedly leading to social withdrawal, reduction in cognitive function, and ability to perform daily tasks including using the internet, driving, and health navigation (24–26). The mechanisms causing disease are unclear but possibly relate to HIV persistence both in the brain and peripheral tissues that are associated with chronic inflammation that may lead to cellular activation. Utilizing a cohort of well-defined human autopsy brain tissue from nVS and VS PWH, we characterized cell activation and its relationship to local viral reservoirs in the brain. Specifically, a higher frequency of astrocytes and myeloid cells harboring activated IFN pathways and production of TNFα and TGF-β1 were observed, demonstrating an activated inflammatory state in the brain. Importantly, we identified that levels of total, intact, and 5′ defective proviruses in the frontal cortex of PWH were associated with increased measures of cellular activation.

In this study we observed an elevated frequency of Mx1-expressing cells in the gray matter of the frontal cortex of VS PWH relative to HIV-seronegative controls, which recapitulates our findings from ART-suppressed SIV-infected NHPs (18). Mx1 production is triggered via the Stat1 pathways following activation of IFNα/β receptors (IFNAR) by type I IFNs (i.e., IFNα/β) (27). Therefore, HIV infection in the brain results in IFN production leading to augmented expression of IFN stimulated genes (including Mx1) in infected and surrounding cells resulting in an elevated state of cellular activation and cognitive function. Type I IFNs including IFNα and β are traditionally associated with anti-viral-mediated effects both *in vitro* and *ex vivo*, and chronically elevated IFN signaling is also associated with the pathogenesis of some neuroinflammatory conditions (28, 29). Furthermore, a series of recent studies using human brain organoid models also observed increased Mx1 and interferon signaling in response to HIV infection despite ART suppression (30–32), supporting our *ex vivo* observations. *In vivo* studies using IFNAR knockout rodent models of neuroHIV have demonstrated an improved effect on cell damage and behavioral measures after knocking down IFN signaling (33), supporting a deleterious role of heightened interferon activation in the brain. Given the role of IFN signaling in mediating viral infection, uncontrolled peripheral viremia in nVS PWH may have a greater impact on the level of Mx1-expressing cells in the brain than local viral persistence alone, particularly in myeloid cells in nVS PWH. These findings support the further assessment of the impact of chronic IFN signaling on brain cells and cognitive outcomes in PWH.

Significantly, we found that the levels of the Mx1 in frontal cortex tissue correlated with levels of total, intact, and 5′ defective HIV proviruses, supporting a mechanism by which the local inflammatory environment influences viral persistence likely through elevated levels of cellular activation. Intact proviruses contain the necessary viral machinery for persistence, viral replication and, in the absence of ART, the generation of infectious virions as demonstrated by *ex vivo* studies from the brain tissue of both NHPs and humans (34). Defective proviruses, as characterized in this study by the IPDA, have been shown to represent replication incompetent viruses that may retain transcriptional activity and even produce select viral proteins that can contribute to cellular activation (35–37). 5′ defective viruses identified by Bruner et al. were defined as being predominantly intact and predicted to be capable of producing the majority of HIV proteins, albeit at a lower level than intact virus (35). Therefore, the association between 5′ defective proviruses in the frontal cortex and Mx1 is unlikely driven by viral replication and instead may reflect the generation of viral transcripts and possible low-level production of viral proteins (37, 38), although these were not specifically assessed in this study. A study of defective proviruses missing the 5′ untranslated region demonstrated that 5′ defective proviruses were able to generate viral transcripts and proteins by using alternative reading frames (39), supporting a role of 5′ defective proviruses in modulating neuroinflammation. Therefore, our *ex vivo* findings from a cohort of autopsy brain tissue support the need for future studies to define the mechanisms governing cell activation in response to 5′ defective proviral DNA in the brain.

VS PWH had a higher level of TNFα-producing cells in the brain, which extends on previous observations in nVS PWH (40, 41), highlighting that even in the presence of ART, a higher proportion of TNFα-producing myeloid cells persists in the CNS. TNFα is a broadly proinflammatory cytokine known to activate and recruit microglia and astrocytes via TNF receptor 1 to sites of inflammation (42, 43). Plasma levels of TNFα are associated with adverse disease progression in untreated PWH (44), and plasma levels of soluble TNF receptor I (a surrogate measure of TNF-α) remain elevated in the plasma of virally suppressed PWH and are associated with HIV mortality independent of CD4+ T-cell counts (45, 46). A previous study in nVS PWH with dementia identified elevated levels of TNFα expression in the brain and astrogliosis (41, 47); however, we did not observe an association between levels of TNFα and the frequency GFAP^+^ astrocytes in our cohort. Myeloid cells were the major producer of TNFα in VS PWH, as was previously observed in nVS PWH (40), and levels appeared to be distributed throughout both white and gray matter of the brain. While the frequency of total myeloid cells did not differ between groups, the frequency of cells expressing TNFα and Mx1 were higher in the brain of PWH, potentially reflecting cell activation rather than greater cellular recruitment. Similarly, the level of cells expressing the astrocyte activation marker GFAP+ and specifically GFAP+ cells expressing TNF-α were also higher in both white and gray matter of VS PWH, supporting activation of astrocytes throughout the frontal cortex of the brain. Given the role of astrocytes in maintaining homeostasis in the brain, chronic activation of astrocytes may lead to cell dysfunction and/or adverse cognitive outcomes in the brain of PWH. Furthermore, although neurons are not directly infected by HIV, astrocyte and microglial activation may have deleterious bystander effects on neurons through both release of viral proteins including tat and nef or through generation of proinflammatory cytokines (31, 48). We have previously demonstrated that HIV genomes and the viral protein p24 were detected in myeloid cells in the brain that may drive cellular activation (10, 21). However, here, we did not find an association between the levels of the viral reservoirs (cell associated HIV DNA) and measures of TNFα^+^ myeloid cells. These findings may suggest that TNFα production in the brain is not solely due to local viral persistence in the brain; however, additional studies are required to define the mechanistic drivers of TNFα in the brain of virally suppressed PWH.

Interestingly, expression of TGF-β1 was higher in the frontal cortex of VS and nVS PWH, when compared to seronegative controls, reflecting our previous findings in SIV-infected ART-suppressed NHPs (18), and supports heightened immunoregulatory signaling in the brain during chronic HIV. TGF-β1 is a multifunctional immunosuppressive cytokine that can impact cell activation, proliferation, and apoptosis and has been demonstrated to activate astrocytes and promote glial scar formation in the brain following secretion by activated myeloid cells (49). The formation of fibrotic scars in the CNS has been implicated in neurological disorders and vascular dementia (50). Similarly, in lymph node tissue, TGF-β1-mediated fibroblast activation and the resulting fibrosis may facilitate local HIV replication and persistence and inflammation (51, 52). Furthermore, TGF-β1 has been shown to control HIV reservoir size, at least in the gut, via targeting cell proliferation and inducing apoptosis (53). Heightened TGF-β1^+^ cells in the brain of nVS and VS PWH are also possibly a result of a direct response to heightened cellular activation and the proinflammatory environment observed in both nVS and VS PWH. While we did not observe an association between HIV proviral DNA and TGF-β1 expression, other factors including localized viral protein expression (54), systemic inflammation, and gut damage as seen in NHP models (18) may contribute to elevated TGF-β1 expression in the brain. The impact of gut dysfunction on neuroinflammation could not be assessed here but should be considered in specialized studies.

Associations between the level of proviral genomes and measures of neuroinflammation were limited to Mx1, with no correlation found between total, intact, or 3′ or 5′ defective HIV DNA and TNFα or TGF-β1. Therefore, it is important to recognize that other factors peripheral to the brain may contribute to neuroinflammation such as chronic gut damage, immune activation, and systemic inflammation, which are hallmarks of chronic HIV that are not resolved by ART and are associated with mortality (45). Measures of peripheral inflammation have also been associated with adverse cognitive performance (55–57), and we have previously demonstrated in SIV-infected NHPs a role for gut damage in contributing to neuroinflammation, supporting the contribution of other factors external to the brain on these parameters (18). It is also possible (and likely) that different combinations of ART regimens may have varying impacts on viral suppression and neuroinflammation in the brain. However, this study was not designed to address the impact of specific regimens, and this question may be better addressed in well-controlled SIV-infected NHP studies. Therefore, future studies in larger cohorts of both PWH and/or NHPs and organoid/organotypic brain tissue models are required to delineate alternate mechanisms and widespread impacts of both defective proviral HIV DNA and systemic inflammation on neuroinflammation.

Our study has significant clinical implications regarding the state of cellular activation and inflammation in the brain of VS PWH. We found no association between measures of cellular activation or viral reservoir with age, drug penetrance into the CNS as determined by CNS penetrance score (20), or CD4+ T-cell count (**Supplementary Table S1**), potentially indicating that the markers of cellular activation and inflammation measured were not related to these clinical parameters. This is important, as biological age is associated with cellular and structural changes in the brain including cellular activation (58). Instead, in our cohort, we identified that both local (and likely peripheral) HIV reservoirs and related systemic inflammation are contributors to cellular activation in the brain. Understanding changes in cellular activation in the brain of VS PWH may inform targeted therapeutic approaches to limit neuroinflammation and anti-viral signaling in the brain. Furthermore, targeting HIV DNA and/or related transcription of intact and even 5′ defective viruses by transcriptional inhibitors or other novel targets silencing the viral reservoir may assist in limiting cellular activation in brain parenchyma. Therefore, approaches either directly or indirectly targeting cellular activation and inflammation in the brain may be required to improve brain health and function.

Together, in this study, we demonstrate the presence of chronic immune activation in the brain of VS PWH and a relationship between proviral HIV in the brain, including specifically 5′ defective proviruses, which may contribute to cellular activation and an underlying pathology in the brain that is not abrogated by ART. These findings offer key evidence for ongoing immune activation and viral persistence, highlighting a need to better understand defective proviruses as a contributor to neuroinflammation and cognitive disorders, which must be considered to improve brain health and cognitive function in VS PWH.

## Data Availability

The raw data supporting the conclusions of this article will be made available by the authors, without undue reservation.
