# Supplementary material for: Neuroinflammation associated with proviral DNA persists in the brain of virally suppressed people with HIV
Source: Front Immunol. 2025 May 21;16:1570692. doi: 10.3389/fimmu.2025.1570692 (PMC12135078; doi:10.3389/fimmu.2025.1570692)
Supplement: Supplementary file 1 [file DataSheet1.docx]

Supplementary Material


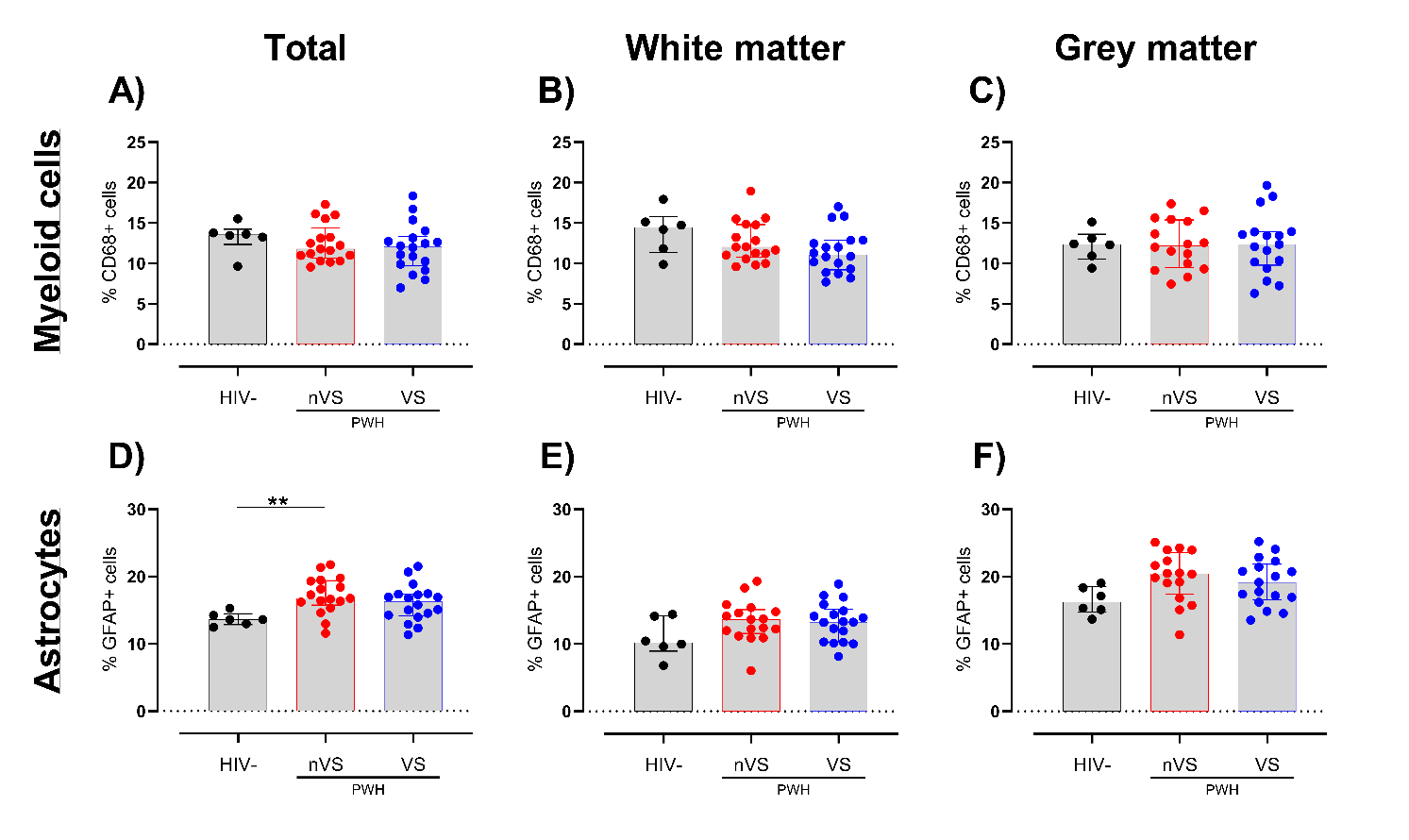


**Supplementary Figure 1. Non-virally suppressed and virally suppressed PWH have a higher frequency of GFAP+ cells in the frontal cortex.** Frequency of (A-C) myeloid cells and (D-F) astrocytes in the total, white matter and grey matter of the frontal cortex from non-virally suppressed (nVS PWH; n=16-17) and virally suppressed people with HIV (VS PWH; n=17-18) and HIV seronegative controls (HIV-; n=6). Data unavailable for n=1 nVS PWH and n=1 VS PWH due to no grey matter region present in tissue section provided. Comparisons made using Kruskal-Wallis test with Dunn’s post-hoc tests (*P<0.05; **P<0.01; ***P<0.001).

| 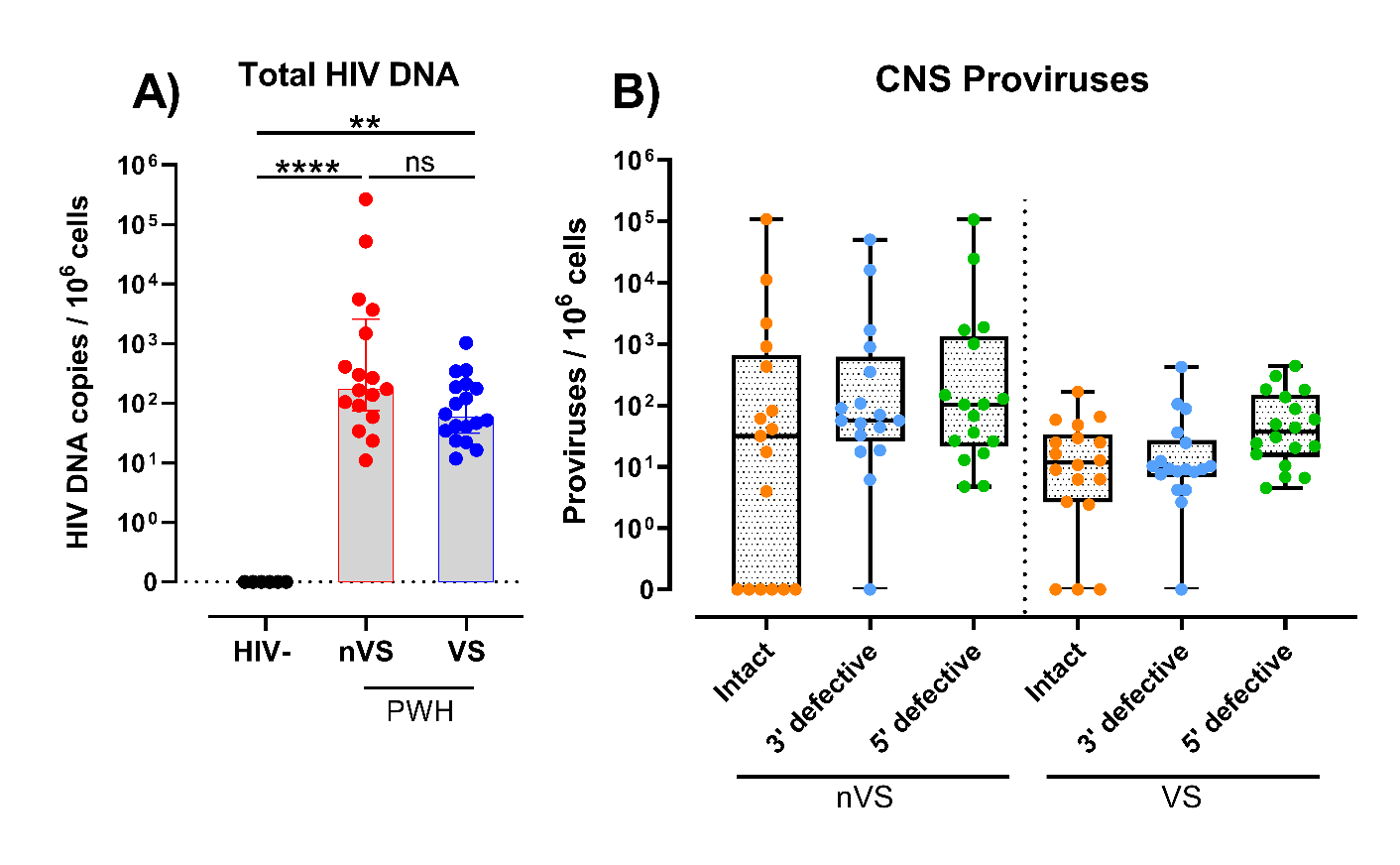 |
| --- |
| **Supplementary Figure 2. HIV DNA persists in the frontal cortex of people with HIV despite viral suppression with ART. (A)** Total HIV DNA copies in human frontal cortex tissue from non-virally suppressed (nVS; n = 17) or virally suppressed (VS; n = 18) people with HIV or HIV-seronegative individuals (HIV-; n = 6) as measured by droplet digital PCR. **(B)** Copy number (standardized to 10^6^ cells) of proviral genomes defined as intact (orange), 3′ defective (light blue) or 5′ defective (green) from frontal cortex tissue from nVS (n = 6) or VS (n = 10) PWH, or HIV- individuals (n = 4) as measured by the IPDA. Comparisons made using Kruskal-Wallis test with Dunn’s post-hoc tests (*P<0.05; **P<0.01; ***P<0.001,***P<0.0001). |

| **Supplementary Table 1. Association between age and immune phenotype in the frontal cortex of PWH** | | | | |
| --- | --- | --- | --- | --- |
|  | Age | | | |
|  | Non-virally suppressed | | Virally suppressed | |
|  | Rho | P value | Rho | P value |
| Mx1 | -0.201 | 0.436 | 0.111 | 0.662 |
| TNFα | 0.371 | 0.143 | 0.332 | 0.178 |
| TGF-β1 | -0.021 | 0.938 | 0.092 | 0.716 |
| CD68 | -0.097 | 0.710 | 0.147 | 0.560 |
| GFAP | -0.130 | 0.617 | 0.276 | 0.268 |
| GFAP: glial fibrillary acidic protein, Mx1: MX dynamin like GTPase, TGF-β1: transforming growth factor beta, TNFα: tumour necrosis factor alpha  P value and rho determined by non-parametric Spearman correlation (P<0.05 statistically significant) | | | | |
